# Supplementary material for: Biosensor and machine learning-aided engineering of an amaryllidaceae enzyme
Source: Nat Commun. 2024 Mar 7;15:2084. doi: 10.1038/s41467-024-46356-y (PMC10920890; doi:10.1038/s41467-024-46356-y)
Supplement: Supplementary file 1 — Supplementary Information [file 41467_2024_46356_MOESM1_ESM.pdf]

Biosensor and Machine Learning-Aided Engineering of an Amaryllidaceae Enzyme

Simon d'Oelsnitz* [1,6], Daniel J. Diaz[2,3], Wantae Kim[4], Daniel J. Acosta[1], Tyler L. Dangerfield[1], Mason W. Schechter[1], Matthew B. Minus[5], James R. Howard[2], Hannah Do[1], James M. Loy[1], Hal S. Alper[4], Y. Jessie Zhang[1], Andrew D. Ellington[1]

[1]Department of Molecular Biosciences, University of Texas at Austin, Austin, TX, 78712, USA
[2]Department of Chemistry, University of Texas at Austin, Austin, TX, 78712, USA
[3]Institute for Foundations of Machine Learning, University of Texas at Austin, Austin, TX, 78712, USA
[4]McKetta Department of Chemical Engineering, University of Texas at Austin, Austin, TX, 78712, USA
[5]Department of Chemistry, Prairie View A&M University, 100 University Dr, Prairie View, TX 77446
[6]Present Address: Synthetic Biology HIVE, Department of Systems Biology, Harvard Medical School, Boston, MA, 02115, USA

Supplementary Information:

Supplementary Figures 1 - 16

Supplementary Tables 1 - 7

Supplementary Discussion 1

Supplementary Figures

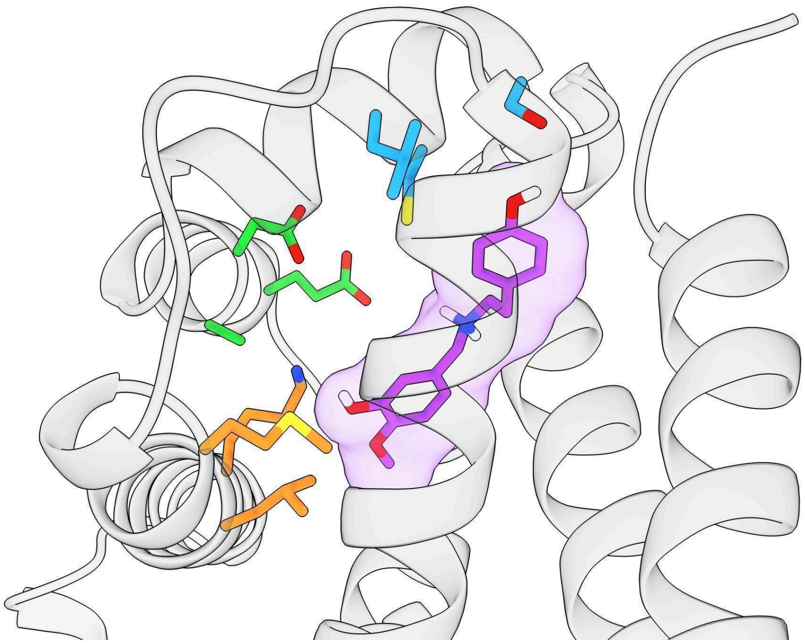

Supplementary Figure 1: Structural depiction of RamR library designs.
The structure of RamR (PDB: 3VVX) was docked with 4'-O-methylnorbelladine (purple) using the GNINA1.0 docking software. The side chains of residues targeted for site-saturation mutagenesis are color coded as follows. Orange: K63, L66, M71; Green: E120, A123, D124; Blue: L133, C134, S137.

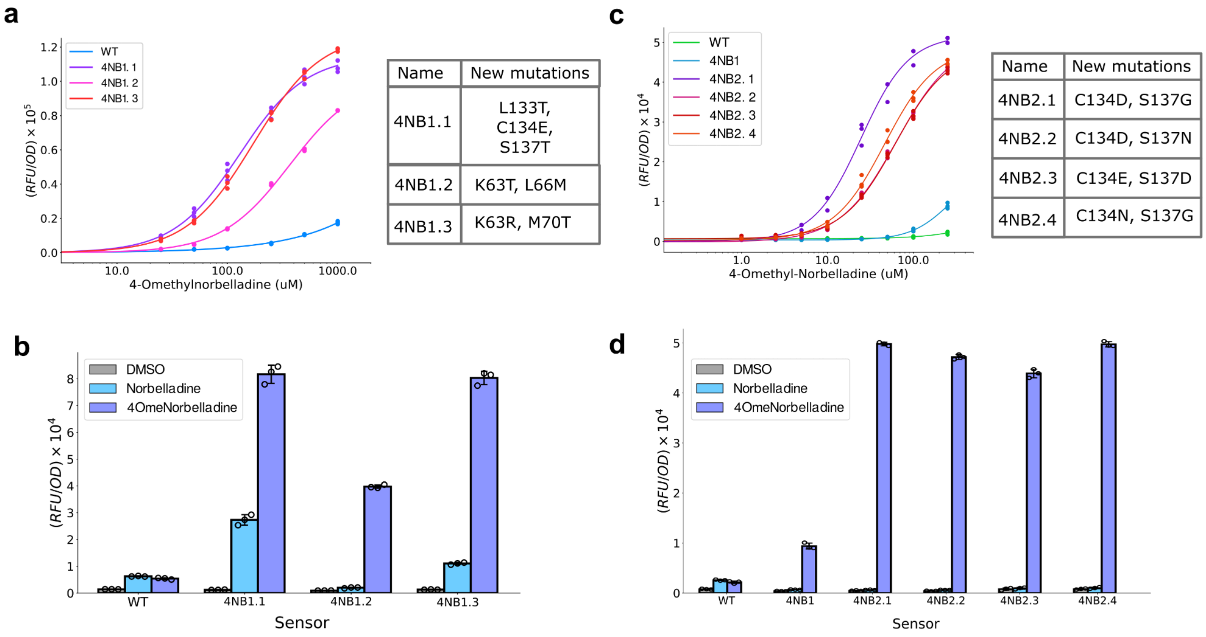

Supplementary Figure 2: The sensitivity and selectivity of RamR mutants evolved for 4'-O-methylnorbelladine. (a) Dose response measurements and genotypes of generation one RamR sensors. (b) Selectivity of generation one RamR sensors. (c) Dose response measurements and genotypes of generation two RamR sensors. (d) Selectivity of generation two RamR sensors. For measurements performed in (b, d), cells were cultured with 100 uM of 4'-O-methylnorbelladine. Experiments presented in panels a-d were conducted in biological triplicate (n=3). Error bars represent the S.D. +/- the mean.

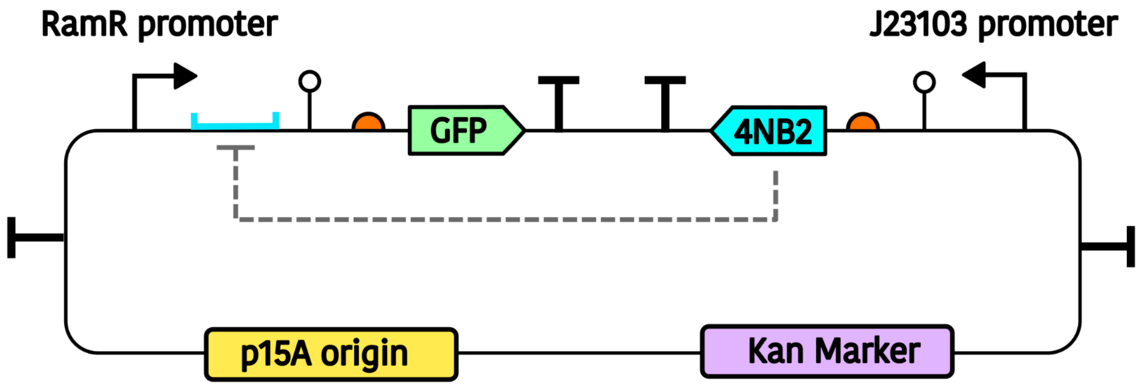

Supplementary Figure 3: Plasmid architecture for the one-plasmid 4'-O-methylnorbelladine reporter system. Standard SBOL glyphs apply. The 4'-O-methylnorbelladine-responsive RamR variant (4NB2) is displayed in cyan and represses the RamR promoter.

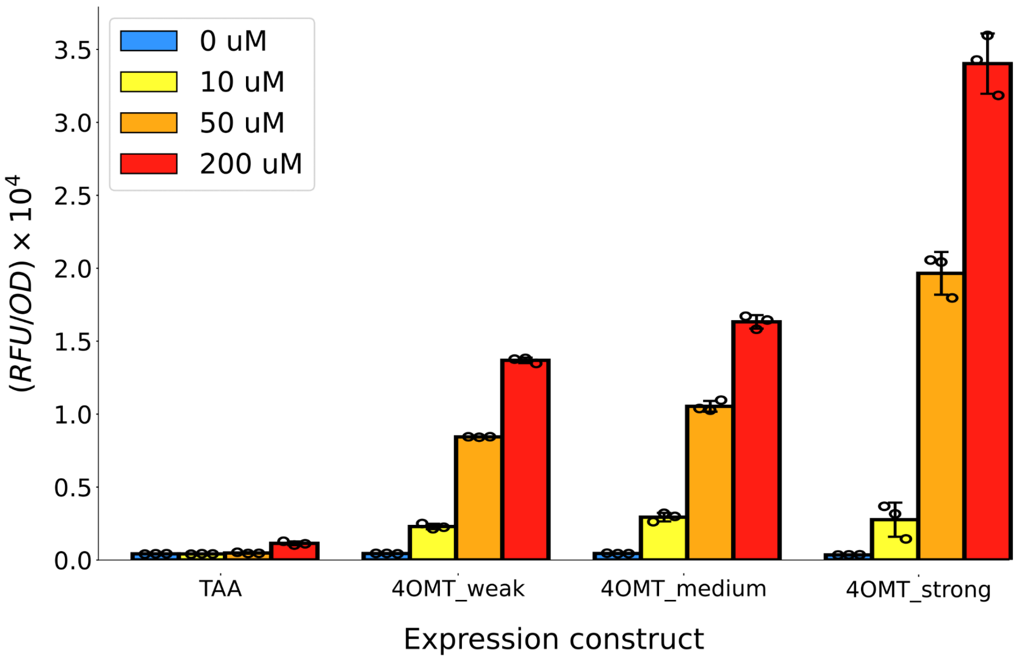

Supplementary Figure 4: Fluorescent response of *E. coli* cells bearing the 4NB2 sensor plasmid with varying levels of Nb4OMT expression and precursor (norbelladine) supplementation. TAA represents an empty plasmid control in place of the Nb4OMT gene. Measurements were performed in biological triplicate (n=3) and error bars represent the S.D. +/- the mean.

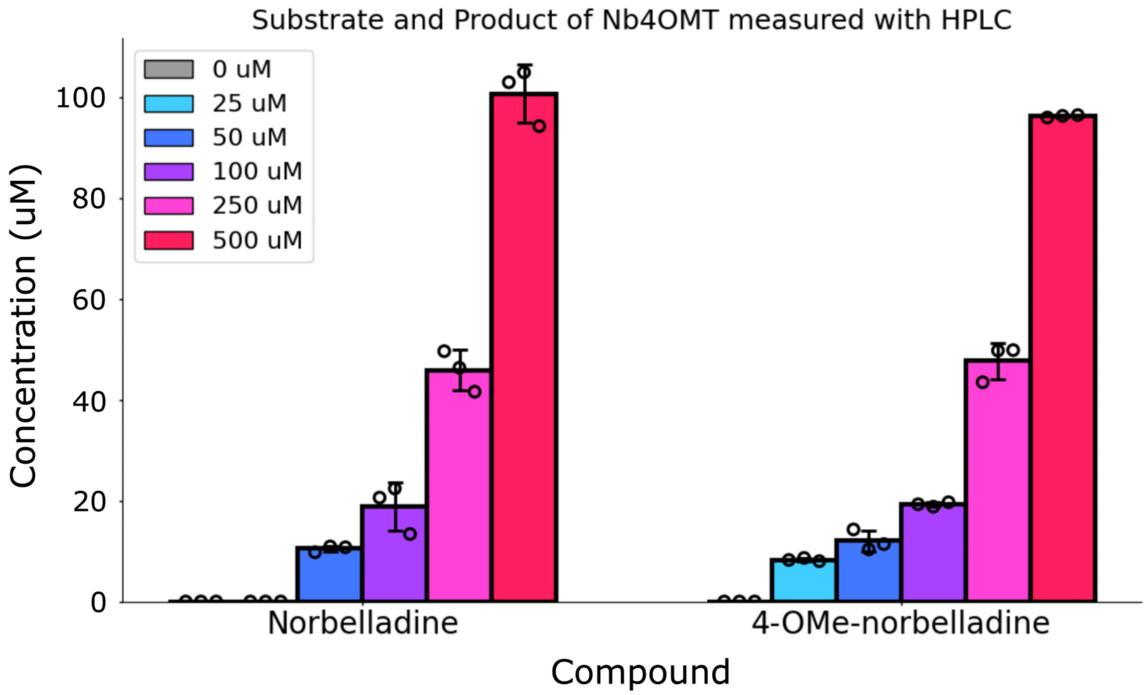

Supplementary Figure 5: Substrate and product of *in vivo* Nb4OMT reaction measured with HPLC. *E. coli* cells expressing the wild-type Nb4OMT enzyme were cultured with varying amounts of norbelladine for 18 hours and the concentrations of norbelladine and 4'-O-methylnorbelladine were subsequently measured via HPLC. Colors denote the concentration of norbelladine supplemented in the culture media during the reaction. Some amount of norbelladine is assumed to be lost during the reaction due to oxidation and degradation. Measurements were performed in biological triplicate (n=3) and error bars represent the S.D. +/- the mean.

a

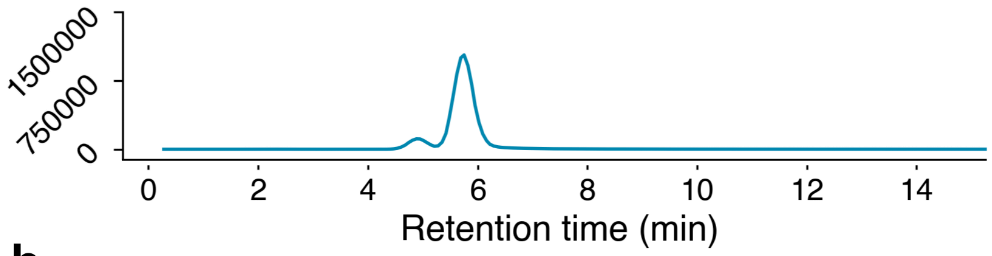

b

Area Major Product	Area Minor Product	Major:Minor Ratio
$1.23*10^8$	$1.28*10^7$	9.59

Supplementary Figure 6: LC/MS analysis of the Nb4OMT-catalyzed *in vivo* reaction. *E. coli* cells (n=1) expressing wild-type Nb4OMT were cultured for 24 hours with 500 µM of norbelladine and the culture supernatant was filtered and analyzed using LC/MS. (**a**) Ion-extracted chromatogram of the reaction product. The y-axis indicates the ion count. The 274.1438 m/z ratio was used for extraction, since this m/z ratio is expected from all single methylated norbelladine products. (**b**) Statistics covering relative ion counts for the minor and major products.

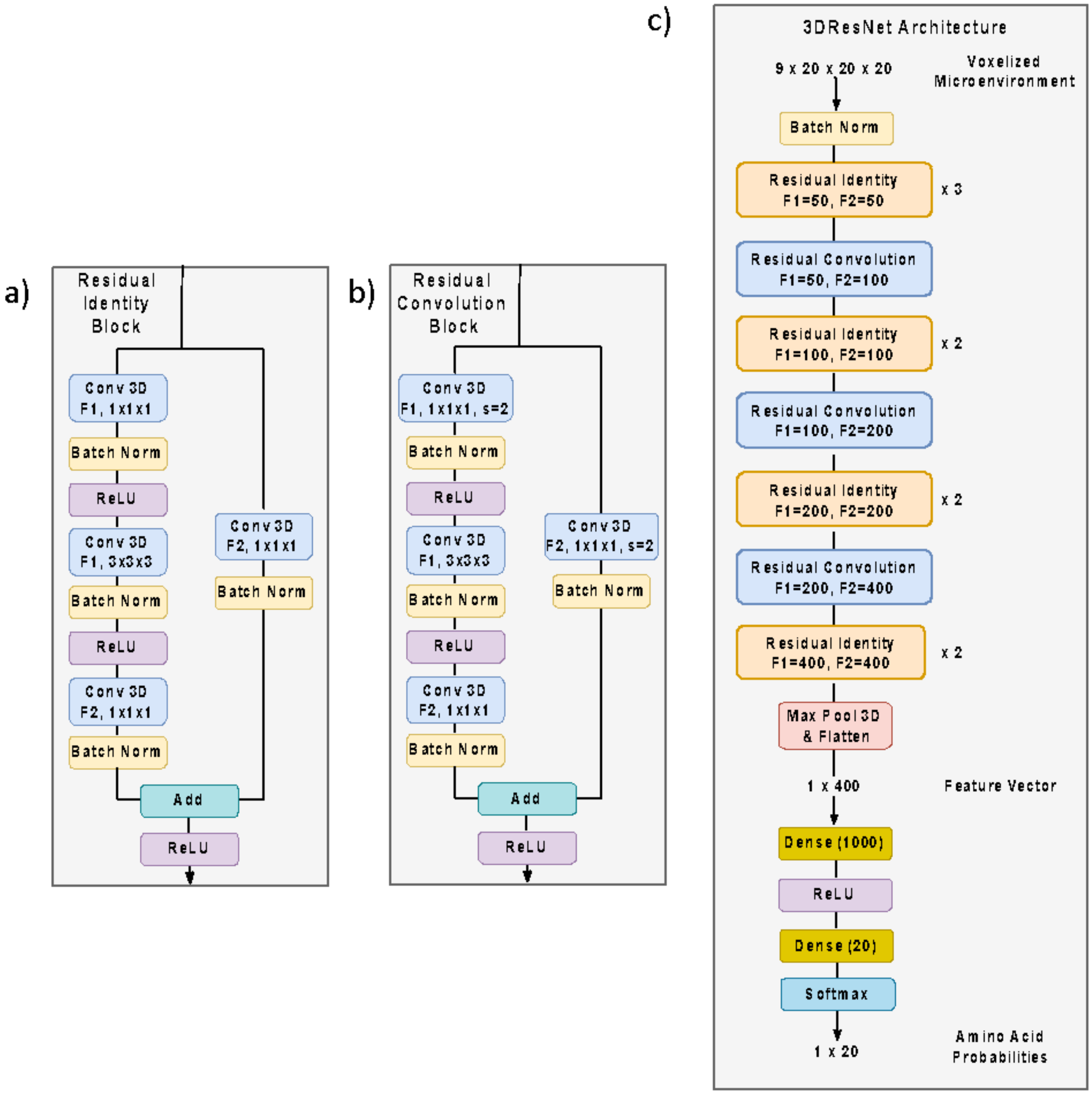

Supplementary Figure 7. Architecture details of 3DResNet: A) Residual Identity block B) Residual Convolutional block C) Full architecture of 3DResNet describing how a voxelized microenvironment (batch_size, 9 channels, 20x, 20y, 20z) is fed through the 3D residual feature extractor and converted into a 400-dimensional feature vector and then passed into a classifier to generate 20 amino acid probability distribution. Conv 3D: 3D Convolution layer, convolution kernel dimensions: 1x1x1 or 3x3x3, F1 and F2 are the number of feature maps generated by the convolution layer. S=2: stride of 2 used for that convolution layer else S=1 was used, ReLU: Rectified Linear Unit, Batch Norm: 3D Batch Normalization layer. Each convolution layer had L2 weight decay regularization set to 0.001. Batch normalization was instantiated with default hyperparameters.

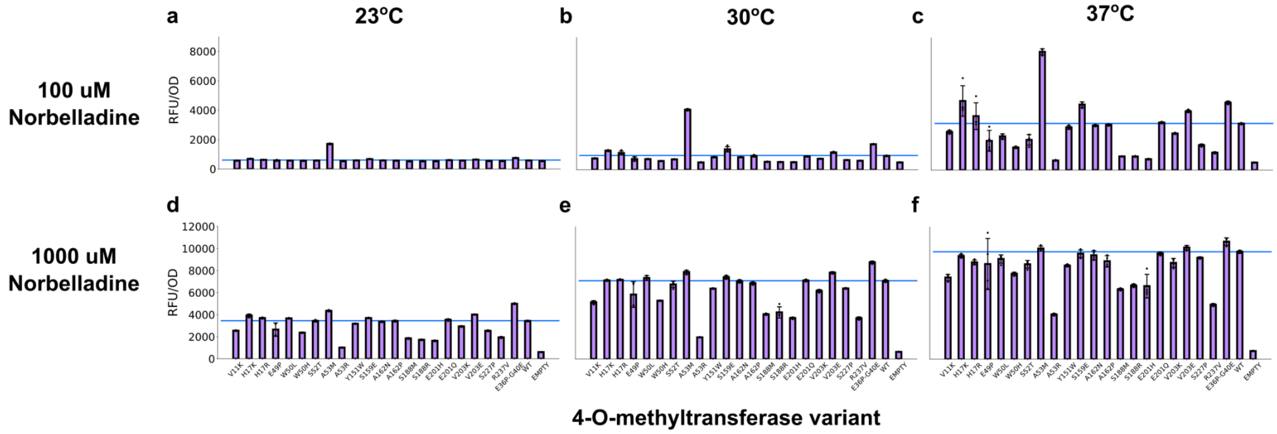

Supplementary Figure 8: Fluorescent response of cells containing single 4OMT variants across two precursor supplementation concentrations (100 µM or 1000 µM) and three fermentation temperatures (23ºC, 30ºC, 37ºC) . In panels **a-c**, 100 µM of norbelladine was supplemented in the media, whereas for panels **d-f**, 1000 µM was supplemented. Culturing temperatures were set at 23ºC, 30ºC, and 37ºC for panels, **a** & **d**, **b** & **e**, and **c** & **f**, respectively. Measurements were performed in biological triplicate (n=3) and error bars represent the S.D. +/- the mean.

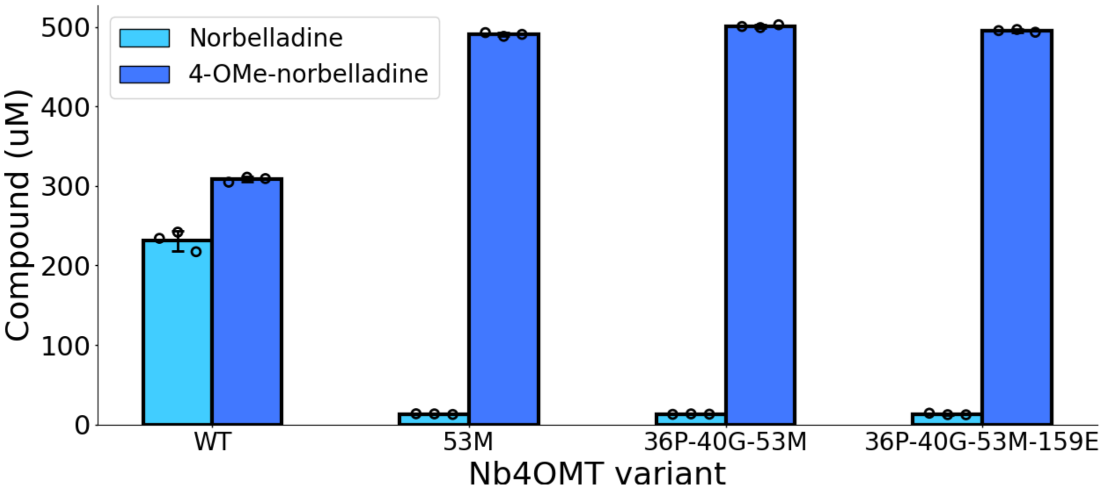

Supplementary Figure 9: HPLC-measured substrate and product concentrations resulting from *in vivo* reactions with Nb4OMT variants. Reactions were carried out within *E. coli* cells cultured for 24 hours at 37ºC with 500 µM of norbelladine supplemented in the media. The resulting culture supernatant was filtered and compound concentrations were determined using HPLC. Mutations relative to the wild-type Nb4OMT sequence are labeled (for example, “53M”). Measurements were performed in biological triplicate (n=3) and error bars represent the S.D. +/- the mean.

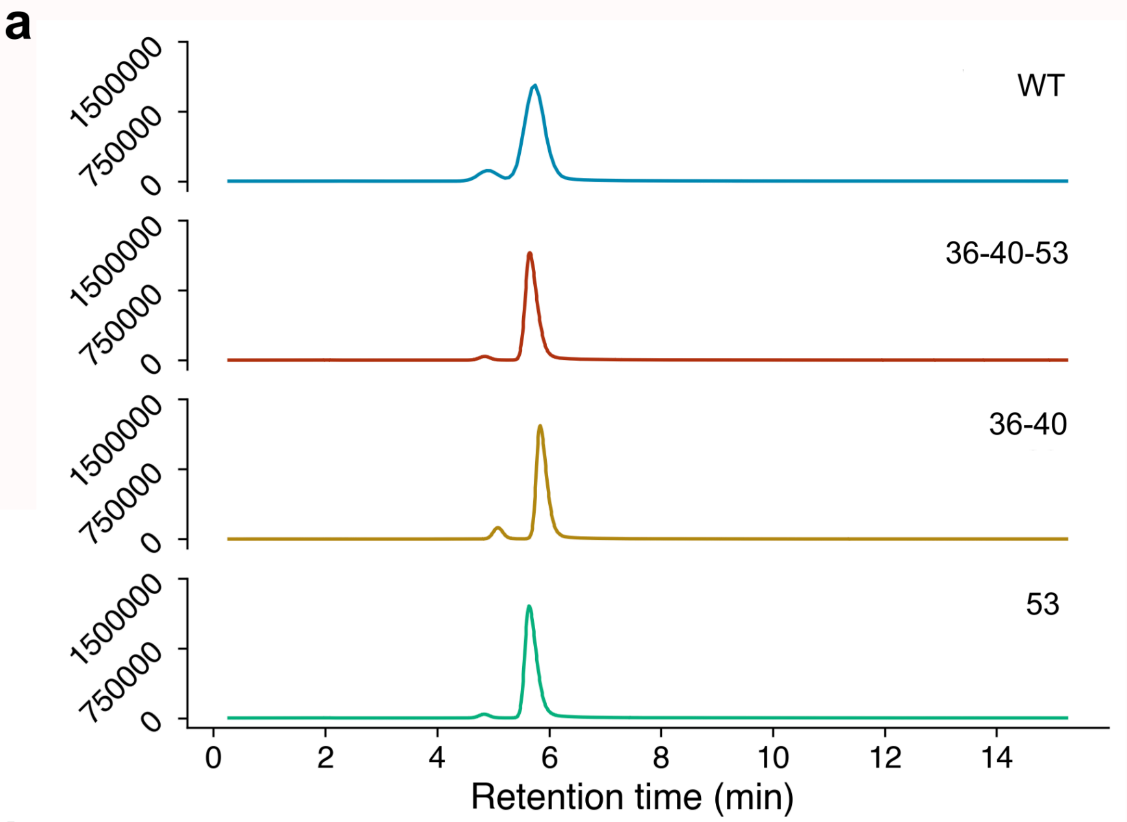

b

Variant	Area Major Product	Area Minor Product	Major:Minor Ratio
WT	$1.28*10^8$	$1.23*10^7$	9.59
36-40-53	$1.85*10^7$	$5.92*10^5$	31.32
36-40	$1.71*10^7$	$1.63*10^6$	10.50
53	$1.90*10^7$	$5.79*10^5$	32.75

Supplementary Figure 10: LC/MS analysis of the Nb4OMT variant-catalyzed *in vivo* reaction. *E. coli* cells (n=1) expressing Nb4OMT variants were cultured for 24 hours with 500 µM of norbelladine and the culture supernatant was filtered and analyzed using LC/MS. (**a**) Ion-extracted chromatogram of the reaction product. The y-axis represents ion counts. The 274.1438 m/z ratio was used for extraction, since this m/z ratio is expected from all single methylated norbelladine products. (**b**) Statistics covering relative ion counts for the minor and major products. Variant name to mutation mapping is as follows, WT: the natural Nb4OMT sequence; 53: A53M; 36-40: E36P + G40E; 36-40-53: E36P + G40E + A53M.

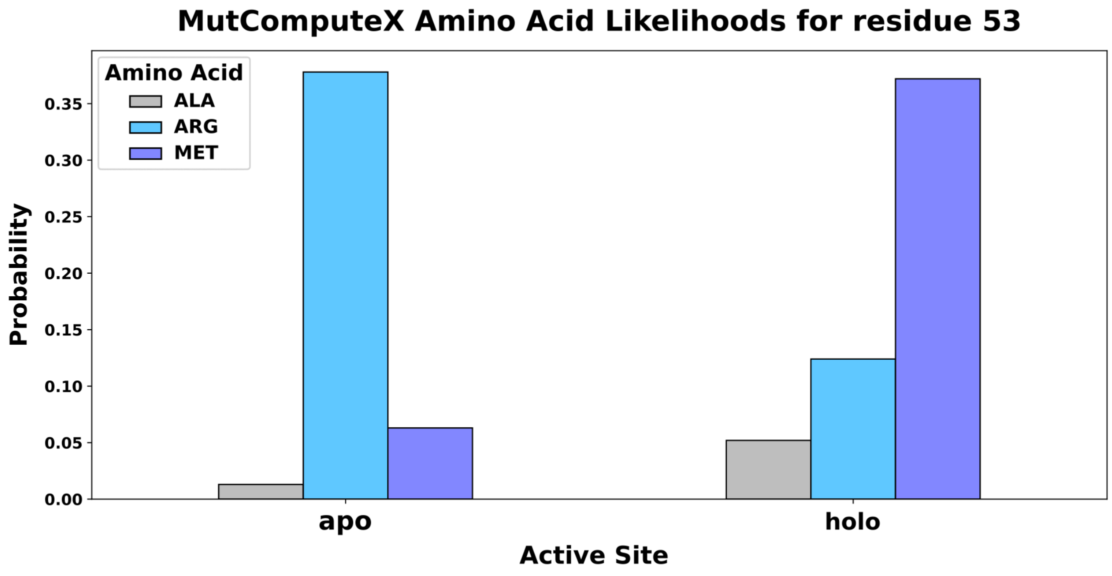

Supplementary Figure 11: MutComputeX predictions for the 53 position with and without ligand docking. AF and Exp indicate that either the AlphaFold2 structure model or the experimentally-determined crystal structure were passed as inputs to MutComputeX, respectively. “Apo” indicates that the structure was not docked with any norbelladine using GNINA1.0, where “Holo” indicates that the structure was docked.

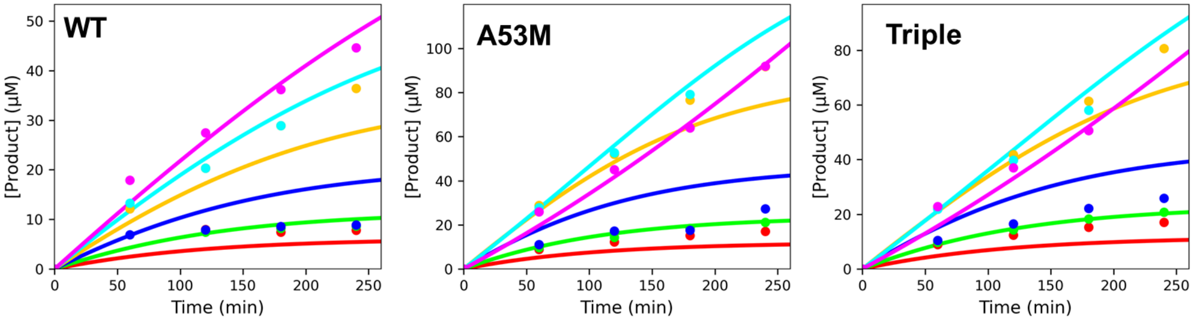

Supplementary Figure 12: Steady state kinetics of Nb4OMT variants. Steady state experiments were set up as described in the methods section. Plots show the best fits by simulation in KinTek Explorer for the concentration of product at each substrate concentration as a function of time. Red, green, blue, yellow, cyan, and purple lines show data at 15.625, 31.25, 62.5, 125, 250, and 500 μM of substrate, respectively.

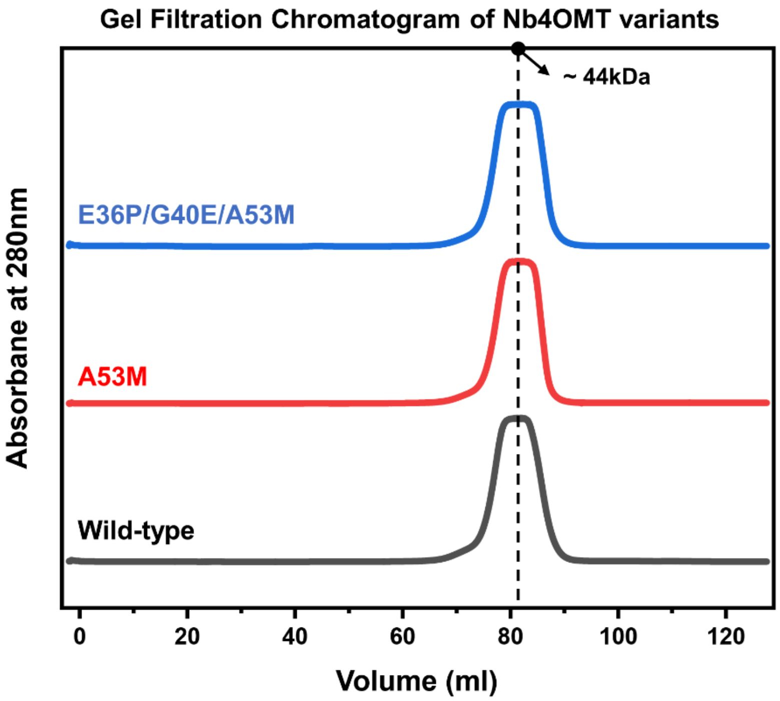

Supplementary Figure 13: Size exclusion chromatography profiles of purified Nb4OMT variants.

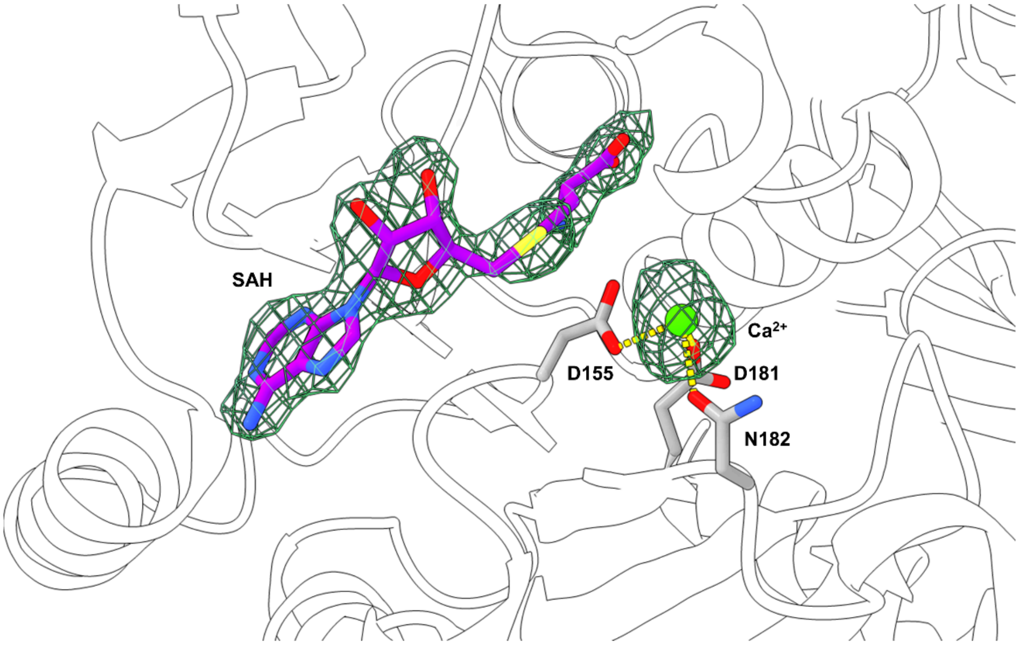

Supplementary Figure 14: Omit Fo-Fc map (contoured at 3.0σ) shown as green meshes superimposed on the stick and sphere model of S-adenosyl-L-homocysteine (purple) and Ca^{2+} ion.

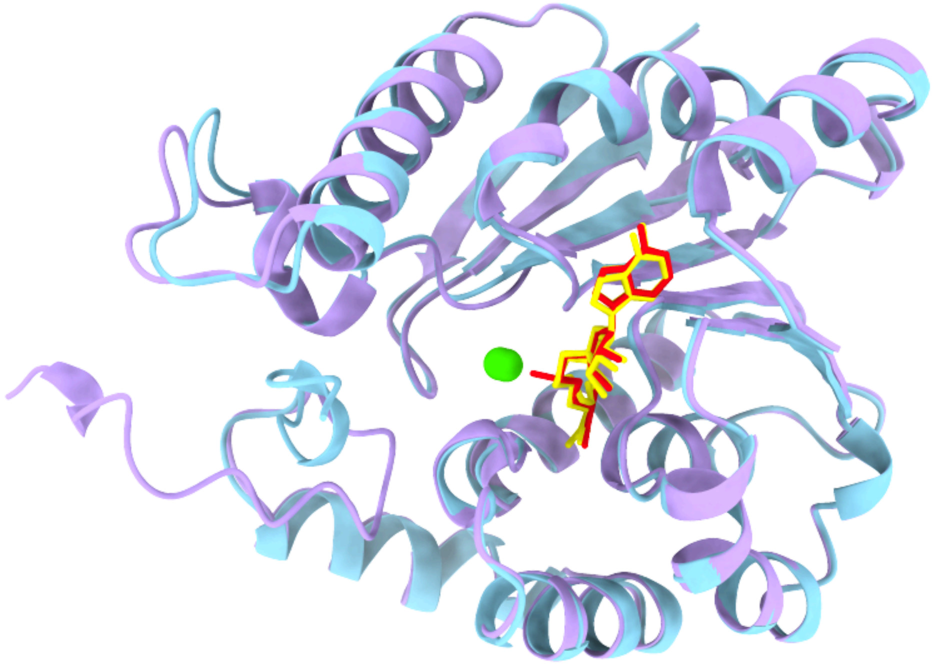

Supplementary Figure 15: Alignment of the crystal structure and AlphaFill model of Nb4OMT
Color coding is as follows: AlphaFill model: purple; Crystal structure: blue; Ca^{2+} ions: green; SAH cofactor in the AlphaFill model: red; SAH cofactor in the Crystal structure: yellow. The norbelladine substrate was not included in the AlphaFill model.

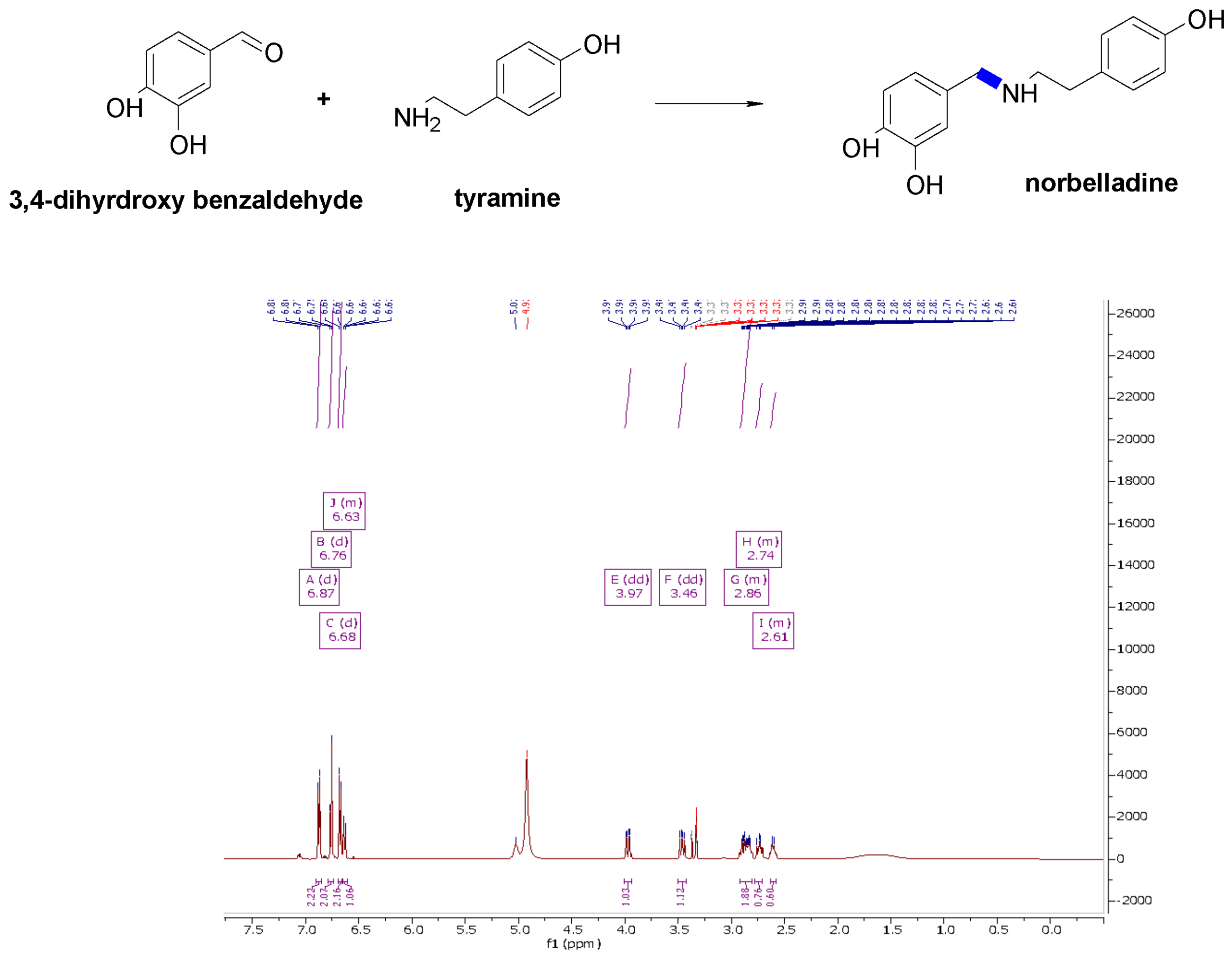

Supplementary Figure 16: Chemical synthesis and NMR validation of norbelladine.

NMR spectra were taken on the 500 MHZ Bruker prodigy at University of Texas at Austin. NMR solvents (CD3OD). ^{1}H NMR (500 MHz, MeOD) δ 6.87 (d, *J* = 8.2 Hz, 2H), 6.76 (m, *J* = 7.8 Hz, 2H), 6.67 (d, *J* = 8.2 Hz, 2H), 6.63 (d, *J* = 8.1 Hz, 1H), 3.97 (q, *J* = 5.6 Hz, 1H), 3.46 (q, *J* = 7.6 Hz, 1H), 2.87 (m, *J* = 4.6 Hz, 2H), 2.74 (m, *J* = 7.6 Hz, 1H), 2.61 (m, *J* = 3.5 Hz, 1H). Characterization was in accordance with previously reported literature.[1]

Supplementary Tables

4NB ligand supplemented into media(uM)	HPLC measurement	HPLC error	4NB2.1 biosensor measurement	4NB2.1 biosensor error
0	4.25	7.22	183.85	10.94
1	2.96	1.28	200.81	4.13
2.5	8.53	10.15	240.62	11.25
5	17.02	10.57	395.69	19.35
10	22.47	9.78	944.28	46.35
25	33.94	9.7	5648.44	409.69
50	50.89	0.82	21194.72	716.82
100	122.04	14.18	43800.94	1702.33
250	260.25	1.5	64506.12	3283.8
500	523.06	3.62	67029.6	2473.36
1000	1058.36	7.48	62040.21	1175.72

Supplementary Table 1: 4’-O-methylnorbelladine measurements made with HPLC and the 4NB2.1 biosensor. HPLC values represent the measured AUC fit to a standard curve. Biosensor measurements represent Relative Fluorescence Units divided by OD600 (RFU/OD). Error represents the standard deviation +/- the mean.

GNINA1.0 docking results				
ligand	minimized Affinity (Kcal/mol)	Minimised RMSD (Angstrom)	CNN Score (probability)	CNN Affinity (pK)
S-adenosyl-Homocysteine	-7.918	0.557	0.835	5.851
Norbelladine	-7.261	0.162	0.824	5.303

Supplementary Table 2: GNINA1.0 docking metrics for S-adenosyl-Homocysteine(SAH) and Norbelladine.

Mutant Alias	Genotype
17-203	H17K, V203E
17-36	H17K, E36P, G40E
17-159	H17K, S159E
36-203	E36P, G40E, V203E
17-53	H17K, A53M
17-53-203	H17K, A53M, V203E
17-53-159	H17K, A53M, S159E
53-159	A53M, S159E
53-203	A53M, V203E
36-53	E36P, G40E, A53M
36-53-203	E36P, G40E, A53M, V203E
36-53-159	E36P, G40E, A53M, S159E

Supplementary Table 3: Genotypes and aliases of combinatorial Nb4OMT mutants

Data collection	
Space group	$P2_1$
Cell dimensions	
a, b, c (Å)	95.97, 79.58, 98.38
α, β, γ (°)	90.00, 105.62, 90.00
Resolution (Å)	50.00-2.40 (2.44-2.40)*
Rsym/ Rpim	0.163(0.441)/0.095(0.278)
CC ½ ^{ϒ}	0.966 (0.778)
I / σ	5.6 (1.6)
Completeness (%)	99.3 (93.9)
Redundancy	3.8 (3.2)
**Refinement**	
Resolution (Å)	47.692 – 2.398 (2.483 – 2.398)
No. reflections	55735 (5310)
R_{work}	0.1891 (0.2357)
$R_{free}^{\pm}$	0.2489 (0.3285)
**No. atoms**	11657
Protein	11349
Ligand/ion	9
Water	299
**B-factors (Å^2)**	
Protein	23.1
Ligand/ion	22.5
Water	24.6
**R.m.s. deviations**	
Bond lengths (Å)	0.0023
Bond angles (°)	0.58
**Ramachandran plot**	
Favored	97.31%
Allowed	2.69%
Outliers	0.00%
**Molprobity score**	1.42 / 99th percentile

*Values for the corresponding parameters in the outermost shell in parenthesis.
$^{ϒ}CC_{1/2}$ is the Pearson correlation coefficient for a random half of the data, the two numbers represent the lowest and highest resolution shell respectively.
$^{\pm}R_{free}$ is the R_{work} calculated for about 10% of the reflections randomly selected and omitted from refinement.

Supplementary Table 4: X-ray Crystallography Data Collection and Refinement Statistics

a)

Interface Type	Percentage (%)
DNA	0.04
RNA	0.72
Ligand	10.9
Halogen	1.17
Protein	23.98
random core/surface	63.19

b)

Amino Acid	Percentage (%)
ALA	7.84
ARG	5.51
ASN	4.39
ASP	6.01
CYS	1.4
GLN	3.72
GLU	6.68
GLY	7.18
HIS	2.71
ILE	5.6
LEU	9.08
LYS	5.6
MET	2.25
PHE	4.17
PRO	4.48
SER	5.89
THR	5.43
TRP	1.53
TYR	3.79
VAL	6.75

Supplementary Table 5: Dataset composition for training/testing 3DResNet models. Dataset consists of 22,584 protein sequences clustered at 50% sequence similarity from the PDB (as of November 2021). The sequences were sampled to generate a 2,569,256 microenvironment dataset, which was then split 90:10 to generate the training and test set, respectively. A) percentage of the dataset sampled at functional interfaces (within 5 angstroms of a non-protein atom or randomly from the core or surface of proteins. B) Amino acid composition of the dataset.

Model	FP Wild Type Accuracy	Pearson ΔTm	Spearman ΔTm	Pearson ΔΔG	Spearman ΔΔG	ΔTm dataset size	ΔΔG dataset size
3DCNN ensemble	0.628	0.325	0.369	-0.369	-0.412	2719	4889
3DResNet ensemble	0.597	0.367	0.425	-0.408	-0.457	2719	4889
3DResNet 1	0.626	0.332	0.383	-0.381	-0.429	2719	4889
3DResNet 2	0.656	0.405	0.426	-0.421	-0.452	2719	4889
3DResNet 3	0.591	0.378	0.435	-0.423	-0.474	2719	4889

Supplementary Table 6: Wildtype accuracy and Pearson and Spearman correlation metrics with ΔTm and ΔΔG experimental values for point mutations in FireProtDB[2] (as of February 2022). Correlations were calculated with the log odds predicted by the deep learning model for the mutated and wildtype amino acids: log $log\,(\frac{mutAA\_probability}{wtAA\_probability}$). The original MutCompute model is labeled as "3DCNN ensemble", while the MutComputeX model is labeled as "3DResNet ensemble".

>pSens4NB2
TGGTTCGTTGTGATGGCGGTAGGAATGTAATCGTTAATCCGCAAATAACGTAAAAACCCGCT
TCGGCGGGTTTTTTTATGGGGGGAGTTTAGGGAAAGAGCATTTGTCATCCCGTTGAATATGG
CTCGCATCTTATCGAGCATACTATCACGTCGGCGACCACTAGTCAGTTAACGCAAGGGCATGG
GCTGTCGACCTTTGAAAAGTACCTTGACGGCGTATCTTTGCTTTCTATAATGAGTGCTTACTC
ACTCATACAATAGTCAGTCATAAGTCTGGGCTAAGCCCACTGATGAGTCGCTGAAATGCGAC
GAAACTTATGACCTCTACAAATAATTTTGTTTAACGTAAACCTCCGGGTTAATAAGGAGTAAT
TATGGCATCCAAGGGCGAGGAGCTCTTTACTGGCGTAGTACCAATTCTCGTAGAGCTCGATG
GCGATGTAAATGGCCATAAGTTTTCCGTACGCGGCGAGGGCGAGGGCGATGCAACTAACGG
CAAGCTCACTCTCAAGTTTATTTGTACTACTGGCAAGCTCCCAGTACCATGGCCAACTCTCGT
AACTACTCTGACCTATGGCGTACAATGTTTTTCCCGCTATCCAGATCACATGAAGCAACATGA
TTTTTTTAAGTCCGCAATGCCAGAGGGCTATGTACAAGAGCGCACTATTAGCTTTAAGGATGA
TGGCACCTATAAGACTCGCGCAGAGGTAAAGTTTGAGGGCGATACTCTCGTAAATCGCATTG
AGCTCAAGGGCATTGATTTTAAGGAGGATGGCAATATTCTCGGCCATAAGCTGGAGTATAATT
TCAATTCCCATAATGTATATATTACCGCAGATAAGCAAAAGAATGGCATTAAGGCGAATTTTA
AGATTCGCCATAATGTGGAGGATGGCTCCGTACAACTCGCAGATCATTATCAACAAAATACTC
CAATTGGCGATGGCCCAGTACTCCTCCCAGATAATCATTATCTCTCCACTCAATCCGTGCTCT
CCAAAGATCCAAATGAGAAGCGCGATCACATGGTACTCCTGGAGTTTGTAACTGCAGCAGG
CATTACTCATGGCATGGATGAGCTCTATAAGCTCGAGCACCACCACCACCACCACTGATAATC
CAAACCTGTTATATGTTAGCTGAGACTAGTTGGAAGTGTGGCTGTCCTCAAGCGTTTTAGTTC
GTCGGTCAGTTTCACCTGATTTACGTAAAAACCCGCTTCGGCGGGTTTTTGCTTTTGGAGGG
GCAGAAAGATGAATGACTGTCGGCCATTCGATGGTGTCGGGTAGCATAACCCCTTGTGATAC
CTTTGCCATGTTTCAGAAACAACTCTGGCGCATCGGGCTTGGACCAAAACGAAAAAAGGCC
GCTTTCGCGGCCTCTTTTCTGGAATTTGGTACCGAGCTCACAGCGGCGATAGTCAGATAGCT
AGACCGTATGTTACCGAGCCTGCACTCCTCGAATTCTTAGGATTATTACTGCTCTTCGCGCGT
AAGTGCGCGCCACATAGCCTCGAAGCCCAACGCAATGTACTCACCAGCGCGAGCCGGGTCG
CGCGCAGCGAAATCCATAGTCGTCTCAGCAAGCGCCAAGAACAACCCGTCGCCGAAGGCGC
GGTACTCGTCGGACATAAACACCATAAGAACGCCACGGTGGTCGAGGTCGCGTAACTCCGG
GAACATATCATCCGCGCGTTGTTCGGTTTCCTTCGTCAACTTTTCAGAAACCGCCAACTGAC
GAATGGCACGATGGCGAGCTGGGTGGTTCAATCCCCAGCTAATATAACTGTTCCAGATAAAA
CGGGTCATCATCTTAGCGTCAGTAATAGAACGATCCAATTCCATGATCATTGATTGGCACATG
TCCTGGGTCAAATGTAAGTAAAGGGTGTTGATCAACTCATCTTTCGTTGCGAAATAGCGGAA
CAACGTCCCTTCCGCAACTCCCGCATTGCGTGCAATTACAGCGGTACTAGCGGCAATGCCTG
ATTGCGCGATGGCTTGAGTTGCCGCTTCAAGCAATGCCTGCTTTTTGTCCTCAGACTTTGGG
CGAGCAACCATATACTAACCTCCTTCTGATACGTGGTTCCGTTAAACAAAATTATTTGTAGAG
GCCCCATTTCGTCCTTTTGGACTCATCAGGGGTGGTACACACCACCCTATGGGGCTCGTAATT
GCTAGCATAATCCCTAGGACTGAGCTAGCTATCAGGGTACTTTTCAAAGGTCGACAGCCCATG
CCCTTGCGTTCGGCAGGTGTACAATGATACGAGGTAATGAAGATGAAGTCCATACAATCGAT
AGATTGGGACCAAAACGAAAAAAGGGGAGCGGTTTCCCGCTCCCCTCTTTTCTGGAATTTG
GTACCGAGTCGCACCTGATTGCCCGACATTATCGCACGGTGTCTCATCTCTGATAACGCATAT
TGTCGTTAGAACTCGGCGCGGCCGCTCACACTGCTTCCGGTAGTCAATAAACCGGTAAACCA
GCAATAGACATAAGCGGCTATTTAACGACCCTGCCCTGAACCGACGACCGGGTCGAATTTGC
TTTCGAATTTCTGCCATTCATCCGCTTATTATCACTTATTCAGGCGTAGCAACCAGGCGTTTAA
GGGCACCAATAACTGCCTTAAAAAAATTAGAAAAACTCATCGAGCATCAAATGAAACTGCA
ATTTATTCATATCAGGATTATCAATACCATATTTTTGAAAAAGCCGTTTCTGTAATGAAGGAGA
AAACTCACCGAGGCAGTTCCATAGGATGGCAAGATCCTGGTATCGGTCTGCGATTCCGACTC
GTCCAACATCAATACAACCTATTAATTTCCCCTCGTCAAAAATAAGGTTATCAAGTGAGAAAT
CACCATGAGTGACGACTGAATCCGGTGAGAATGGCAAAAGTTTATGCATTTCTTTCCAGACT
TGTTCAACAGGCCAGCCATTACGCTCGTCATCAAAATCACTCGCATCAACCAAACCGTTATT
CATTCGTGATTGCGCCTGAGCGAGACGAAATACGCGGTCGCTGTTAAAAGGACAATTACAAA

CAGGAATCGAATGCAACCGGCGCAGGAACACTGCCAGCGCATCAACAATATTTTCACCTGA
ATCAGGATATTCTTCTAATACCTGGAATGCTGTTTTCCCGGGGATCGCAGTGGTGAGTAACCA
TGCATCATCAGGAGTACGGATAAAATGCTTGATGGTCGGAAGAGGCATAAATTCCGTCAGCC
AGTTTAGTCTGACCATCTCATCTGTAACATCATTGGCAACGCTACCTTTGCCATGTTTCAGAA
ACAACTCTGGCGCATCGGGCTTCCCATACAATCGATAGATTGTCGCACCTGATTGCCCGACAT
TATCGCGAGCCCATTTATACCCATATAAATCAGCATCCATGTTGGAATTTAATCGCGGCCTAGA
GCAAGACGTTTCCCGTTGAATATGGCTCATTTTAGCTTCCTTAGCTCCTGAAAATCTCGATAA
CTCAAAAAATACGCCCGGTAGTGATCTTATTTCATTATGGTGAAAGTTGGAACCTCTTACGTG
CCGATCACGTCTCATTTTCGCCAAAGTTGGCCAGGGCTTCCCGGTATCAACAGGGACACCAG
GATTTATTTATNNTGCGAAGTGATCTTCCGTCACAGGTATTTATTCGGCGCAAAGTGCGTCGG
GTGATGCTGCCAACTTACTGATTTAGTGTATGATGGTGTTTTTGAGGTGCTCCAGTGGCTTCT
GTTTCTATCAGCTGTCCCTCCTGTTCAGCTACTGACGGGGTGGTGCGTAACGGCAAAAGCAC
CGCCGGACATCAGCGCTAGCGGAGTGTATACTGGCTTACTATGTTGGCACTGATGAGGGTGT
CAGTGAAGTGCTTCATGTGGCAGGAGAAAAAAGGCTGCACCGGTGCGTCAGCAGAATATGT
GATACAGGATATATTCCGCTTCCTCGCTCACTGACTCGCTACGCTCGGTCGTTCGACTGCGGC
GAGCGGAAATGGCTTACGAACGGGGCGGAGATTTCCTGGAAGATGCCAGGAAGATACTTAA
CAGGGAAGTGAGAGGGCCGCGGCAAAGCCGTTTTTCCATAGGCTCCGCCCCCCTGACAAGC
ATCACGAAATCTGACGCTCAAATCAGTGGTGGCGAAACCCGACAGGACTATAAAGATACCAG
GCGTTTCCCCCTGGCGGCTCCCTCGTGCGCTCTCCTGTTCCTGCCTTTCGGTTTACCGGTGT
CATTCCGCTGTTATGGCCGCGTTTGTCTCATTCCACGCCTGACACTCAGTTCCGGGTAGGCA
GTTCGCTCCAAGCTGGACTGTATGCACGAACCCCCCGTTCAGTCCGACCGCTGCGCCTTATC
CGGTAACTATCGTCTTGAGTCCAACCCGGAAAGACATGCAAAAGCACCACTGGCAGCAGCC
ACTGGTAATTGATTTAGAGGAGTTAGTCTTGAAGTCATGCGCCGGTTAAGGCTAAACTGAAA
GGACAAGTTTTGGTGACTGCGCTCCTCCAAGCCAGTTACCTCGGTTCAAAGAGTTGGTAGCT
CAGAGAACCTTCGAAAAACCGCCCTGCAAGGCGGTTTTTTCGTTTTCAGAGCAAGAGATTA
CGCGCAGACCAAAACGATCTCAAGAAGATCATCTTATTAATCAGATAAAATATTTCTAGATTT
CAGTGCAATTTATCTCTTCAAATGTAGCACCTGAAGTCAGCCCCATACGATATAAGTTGTAAT
TCTCATGTTAGTCATGCCCCGCGCCCACCGGAAGGAGCTGACTGGGTTGAAGGCTCTCAAG
GGCATCGGTCGAGATCCCGGTGCCTAATGAGTGAGCTAACTTACATTAATTGCGTTGCGCTC
ACTGCCCGCTTTCCAGTCGGGAAACCTGTCGTGCCAGCTGCATTAATGAATCGGCCAACGCG
CGGGGAGAGGCGGTTTGCGTATTGGGCGCCAGGGTGGTTTTTCTTTTCACCAGTGAGACGG
GCAACAGCTGATTGCCCTTCACCGCCTGGCCCTGAGAGAGTTGCAGCAAGCGGTCCACGCT
GGTTTGCCCCAGCAGGCGAAAATCCTGTTTGATGGTGGTTAACGGCGGGATATAACATGAGC
TATCTTCGGTATCGTCGTATCCCACTACCGAGATGTCCGCACCAACGCGCAGCCCGGACTCG
GTAATGGCGCGCATTGCGCCCAGCGCCATCTGATCGTTGGCAACCAGCATCGCAGTGGGAA
CGATGCCCTCATTCAGCATTTGCATGGTTTGTTGAAAACCGGACATGGCACTCCAGTCGCCT
TCCCGTTCCGCTATCGGCTGAATTTGATTGCGAGTGAGATATTTATGCCAGCCAGCCAGACGC
AGACGCGCCGAGACAGAACTTAATGGGCCCGCTAACAGCGCGATTTGCTGGTGACCCAATG
CGACCAGATGCTCCACGCCCAGTCGCGTACCATCTTCATGGGAGAAAATAATACTGTTGATG
GGTGTCTGGTCAGAGACATCAAGAAATAACGCCGGAACATTAGTGCAGGCAGCTTCCACAG
CAATGGCATCCTGGTCATCCAGCGGATAGTTAATGATCAGCCCACTGACGCGTTGCGCGAGA
AGATTGTGCACCGCCGCTTTACAGGCTTCGACGCCGCTTCGTTCTACCATCGACACCACCAC
GCTGGCACCCAGTTGATCGGCGCGAGATTTAATCGCCGCGACAATTTGCGACGGCGCGTGC
AGGGCCAGACTGGAGGTGGCAACGCCAATCAGCAACGACTGTTTGCCCGCCAGTTGTTGTG
CCACGCGGTTGGGAATGTAATTCAGCTCCGCCATCGCCGCTTCCACTTTTTCCCGCGTTTTC
GCAGAAACGTGGCTGGCCTGGTTCACCACGCGGGAAACGGTCTGATAAGAGACACCGGCAT
ACTCTGCGACATCGTATAACGTTACTGGTTTCACATTCACCACCCTGAATTGACTCTCTTCCG
GGCGCTATCATGCCATACCGCGAAAGGTTTTGCGCCATTCGATGGTGTCCGGGATCTCGACG
CTCTCCCTTATGCGACGCGGCCGCGGCATCAGAGCAGATTGTACTGTGTCCTCAA

>Nb4OMT-A53M

ATCCCCCTTACACGGAGGCATCAGTGACCAAACAGGAAAAAACCGCCCTTAACATGGCCCG
CTTTATCAGAAGCCAGACATTAACGCTTCTGGAGAAACTCAACGAGCTGGACGCGGATGAA
CAGGCAGACATCTGTGAATCGCTTCACGACCACGCTGATGAGCTTTACCGCAGCTGCCTCGC
GCGTTTCGGTGATGACGGTGAAAACCTCTGACACATGCAGCTCCCGCAGACGGTCACAGCT
TGTCTGTAAGCGGATGCCGGGAGCAGACAAGCCCGTCAGGGCGCGTCAGCGGGTGTTGGC
GGGTGTCGGGGCGCAGCCATGACCCAGTCACGTAGCGATAGCGGAGTGTATACTGGCTTAAC
TATGCGGCATCAGAGCAGATTGTACTGAGAGTGCACCGGTGTGAAATACCGCACAGATGCGT
AAGGAGAAAATACCGCATCAGGCGCTCTTCCGCTTCCTCGCTCACTGACTCGCTGCGCTCGG
TCGTTCGGCTGCGGCGAGCGGTATCAGCTCACTCAAAGGCGGTAATACGGTTATCCACAGAA
TCAGGGGATAACGCAGGAAAGAACATGTGAGCAAAAGGCCAGCAAAAGGCCAGGAACCGT
AAAAAGGCCGCGTTGCTGGCGTTTTTCCATAGGCTCCGCCCCCCTGACGAGCATCACAAAA
ATCGACGCTCAAGTCAGAGGTGGCGAAACCCGACAGGACTATAAAGATACCAGGCGTTTCC
CCCTGGAAGCTCCCTCGTGCGCTCTCCTGTTCCGACCCTGCCGCTTACCGGATACCTGTCCG
CCTTTCTCCCTTCGGGAAGCGTGGCGCTTTCTCATAGCTCACGCTGTAGGTATCTCAGTTCGG
TGTAGGTCGTTCGCTCCAAGCTGGGCTGTGTGCACGAACCCCCCGTTCAGCCCGACCGCTG
CGCCTTATCCGGTAACTATCGTCTTGAGTCCAACCCGGTAAGACACGACTTATCGCCACTGGC
AGCAGCCACTGGTAACAGGATTAGCAGAGCGAGGTATGTAGGCGGTGCTACAGAGTTCTTGA
AGTGGTGGCCTAACTACGGCTACACTAGAAGGACAGTATTTGGTATCTGCGCTCTGCTGAAG
CCAGTTACCTTCGGAAAAAGAGTTGGTAGCTCTTGATCCGGCAAACAAACCACCGCTGGTA
GCGGTGGTTTTTTTGTTTGCAAGCAGCAGATTACGCGCAGAAAAAAAGGATCTCAAGAAGA
TCCTTTGATCTTTTCTACGGGGTCTGACGCTCAGTGGAACGAAAACTCACGTTAAGGCCCTC
TCCAAGACCGAGCCATCAACAAAGCGTCTCGCTGAGGTTTCATGGAGCCTCTGGTTCATCTC
CGGCAATTAAAAAGCGGCTAACCACGCCGCTTTTTTTACGTCTGCAGGAACGGGCTGTCG
ACCTTTGAAAAGTTCGTTTACCGCTAGCTCAGTCCTAGGTACAATTACAGCCATCGTACGAGC
CCTGGCTGAGCACAGCTGTCACCGGATGTGCTTTCCGGTCTGATGAGTCCGTGAGGACGAA
ACAGCCTCTACAAATAATTTTGTTTAAACTAGTGAACCACGAGGCCTACATATGGGTGCTTCA
ATTGATGACTACTCCTTGGTACATAAAAACATCTTGCATTCCGAAGATCTGCTGAAATACATT
CTTGAAACCAGTGCATATCCTCGCGAACACGAACAATTAAAGGGTCTGCGTGAGGTTACTGA
AAAGCACGAATGGTCATCCatgTTAGTACCGGCAGACGAAGGTTTATTCCTGTCAATGTTACTG
AAGTTGATGAATGCAAAACGTACTATCGAAATCGGCGTGTACACGGGATACAGCCTGTTAAC
AACGGCATTGGCTTTACCGGAGGATGGTAAAATTACGGCGATCGATGTAAATAAGAGTTATTA
CGAAATCGGATTGCCCTTTATTCAGAAGGCGGGCGTGGAACACAAGATCAACTTTATTGAGT
CGGAAGCGCTTCCCGTGCTGGATCAAATGTTAGAGGAGATGAAGGAGGAGGATTTATACGAC
TACGCTTTTGTGGATGCTGATAAAAGCAATTATGCCAATTACCATGAGCGTCTTGTAAAACTT
GTACGTATCGGTGGTGCCATCCTGTACGACAATACACTGTGGTATGGTTCTGTTGCGTACCCG
GAATACCCCGGTCTGCATCCAGAGGAAGAAGTCGCGCGTCTGAGCTTTCGTAACTTGAATAC
CTTTTTAGCAGCAGATCCTCGCGTAGAAATTAGTCAAGTCTCAATTGGTGATGGCGTGACCAT
TTGTCGCCGCTTGTATTAATAATCCTATCGCCACTTTCAGCCAAAAAACTTAAGACCGCCGGT
CTTGTCCACTACCTTGCAGTAATGCGGTGGACAGGATCGGCGGTTTTCTTTTCTCTTCTCAAC
ACCCTTCGCGTCAACACTTTTCCGCCAAGGAGACGGTTGGTCAGGTTTTCGGGAGGTGTGG
CTGGAAGTTCCTATACTTTCTAGAGAATAGGAACTTCTTTCTAAATACATTCAAATATGTATCC
GCTCATGAGACAATAACCCTGATAAATGCTTCAATAATATTGAAAAAGGAAGAGTATGAGTAT
TCAACATTTCCGTGTCGCCCTTATTCCCTTTTTTGCGGCATTTTGCCTTCCTGTTTTTGCTCAC
CCAGAAACGCTGGTGAAAGTAAAAGATGCTGAAGATCAGTTGGGTGCACGAGTGGGTTACA
TCGAACTGGATCTCAACAGCGGTAAGATCCTTGAGAGTTTTCGCCCCGAAGAACGTTTTCCA
ATGATGAGCACTTTTAAAGTTCTGCTATGTGGCGCGGTATTATCCCGTGTTGACGCCGGGCAA
GAGCAACTCGGTCGCCGCATACACTATTCTCAGAATGACTTGGTTGAGTACTCACCAGTCAC
AGAAAAGCATCTTACGGATGGCATGACAGTAAGAGAATTATGCAGTGCTGCCATAACCATGA
GTGATAACACTGCGGCCAACTTACTTCTGACAACGATCGGAGGACCGAAGGAGCTAACCGC
TTTTTTGCACAACATGGGGGATCATGTAACTCGCCTTGATCGTTGGGAACCGGAGCTGAATG
AAGCCATACCAAACGACGAGCGTGACACCACGATGCCTGCAGCAATGGCAACAACGTTGCG

CAAACTATTAACTGGCGAACTACTTACTCTAGCTTCCCGGCAACAATTAATAGACTGGATGGA<br>GGCGGATAAAGTTGCAGGACCACTTCTGCGCTCGGCCCTTCCGGCTGGCTGGTTTATTGCTG<br>ATAAATCTGGAGCCGGTGAGCGTGGATCGCGCGGTATCATTGCAGCACTGGGGCCAGATGGT<br>AAGCCCTCCCGTATCGTAGTTATCTACACGACGGGGAGTCAGGCAACTATGGATGAACGAAA<br>TAGACAGATCGCTGAGATAGGTGCCTCACTGATTAAGCATTGGTAAGTTGTGATGGCGGTAG<br>GAATGTAATCGTTAATCCGCAAATAACGTAAAAACCCGCTTCGGCGGGTTTTTTTATGGGGG<br>GAGTTTAGGGAAAGAGCATTTGTCATCCCGTTGAATATGGCTCCCTTAACGTGAGGAAGTTC<br>CTATACTTTCTAGAGAATAGGAACTTCTACAGATGGACTTGGGTTGGCGGTTTCAGGAGTCTG<br>CAAAACGTCTGCGACCTGAGCAACAACATGAATGGTCATCGGTTTCCGTGTTTCGTAAAGTC<br>TGGAAACGCGGAAGTCAGCGCCCTGCACCATTATGTTCCGGATCTGCATCGCAGGATGCTGC<br>TGGCTACCCTGTGGAACACCTACATCTGTATTAACGAAGCGCTGGCATTGACCCTGAGTGATT<br>TTTCTCTGGTCCCGCCGCATCCATACCGCCAGTTGTTTACCCTCACAACGTTCCAGTAACCGG<br>GCATGTTCATCATCAGTAACCCGTATCGTGAGCATCCTCTCTCGTTTCATCGGTATCATTACCC<br>CCATGAACAGAA
**Sequence annotation color code map:**<br>LacI<br>p15A origin<br>Kanamycin resistance<br>Terminator<br>RamR-4NB2<br>GFP<br>RamR promoter<br>pBR322 origin<br>Ampicillin Resistance

Supplementary Table 7: Sequences with color-coded annotations of plasmids used in this study.

Supplementary Discussion

Supplementary Discussion 1: Process for the manual curation of Nb4OMT variants

S188:
SER: <0.01 ARG:0.15 MET: 0.12
ARG and/or MET may have the opportunity to interact with the phenol of norbelladine.
Rank: MET > ARG

A53:
ALA: 0.01 MET: 0.37 ARG: 0.38
MutComputeX prefers MET in the presence of ligand/cofactor, and it prefers ARG with no ligand/cofactor. ARG and MET may form either a Cation-pi interaction or a Sulfur-pi interaction with the catechol ring of norbelladine, respectively.
Rank: MET > ARG

E49:
GLU: 0.07 PRO: 0.66
MutComputeX strongly predicts PRO at the end of an alpha helix and the beginning of a loop involved in ligand binding.
RANK: PRO

E201:
GLU: 0.05 GLN: 0.38 ARG: 0.13 HIS: 0.14 LYS: 0.11
MutComputeX predicts to turn this acid into an amide or into a cation. May interact with the docked phenolic ring of Norbelladine. May form cation-pi interaction or pi-pi interactions.
Rank: GLN > HIS > LYS

W50:
TRP: 0.01 ASN: 0.20 LEU: 0.22 HIS: 0.18
MutComputeX strongly dislikes TRP in both chains. Predicts ASN in one and LEU in the other. HIS is also better predicted compared to TRP.
RANK: HIS > ASN > LEU

S52:
SER: 0.08 THR: 0.67
Net strongly predicts mutating to a THR. SER is directly contacting the amine in norbelladine.
Rank: THR

S227:
SER: 0.04 PRO: 0.77
Net strongly predicts a PRO at the end of a beta strand and beginning of a loop. Can potentially form a salt bridge with D58 of the adjacent protomer in the homodimer. In a hydrophobic pocket, so it might also be worth trying LEU.
Rank: PRO > LYS > LEU

A162:
ALA: 0.02 VAL: 0.22 PRO: 0.13 ILE: ARG: 0.08 LYS: 0.7

At the interface of two alpha helices and is semi-solvent exposed.
RANK: VAL > ARG > PRO

R237:
ARG: 0.07 VAL: 0.85
Net strongly predicts a VAL.
Rank: VAL

V11:
VAL: 0.01 LYS: 0.21
Might form a salt bridge with E47.
Rank: LYS

S159:
SER: 0.05 GLU: 0.44
Net strongly predicts GLU.
Rank: GLU

V203:
VAL: 0.02 GLU: 0.17 LYS: 0.21
MutComputeX predicts either a GLU or LYS depending on the protomer. It is worth trying both.
Rank: LYS > GLU

Y151:
TYR: 0.07 TRP: 0.89
MutComputeX strongly predicts mutating to TRP. This is in a hydrophobic pocket in the core of the protein. TRP is a more hydrophobic aromatic..
Rank: TRP > PHE

H17:
HIS: 0.09 ARG: 0.45 LYS: 0.23
At the interface of two protomers in the homodimer complex. Net strongly predicts a cation here.
Rank: ARG > LYS

E36P & G40E:
MutComputeX strongly predicts a PRO at position 36 to cap the alpha helix. However, at position 40 in the crystal structure of the 1SUI homolog there is a GLU that can form a salt bridge with K118. By removing GLU at E36 the salt bridge will be lost. By making the G40E substitution together with E36P, we can preserve the salt bridge and proline cap the alpha helix.

Supplementary references

1. Park, J. B. Synthesis and characterization of norbelladine, a precursor of Amaryllidaceae alkaloid, as an anti-inflammatory/anti-COX compound. *Bioorganic & Medicinal Chemistry Letters* **24**, 5381–5384 (2014).

2. Stourac, J., Dubrava, J., Musil, M., Horackova, J., Damborsky, J., Mazurenko, S., Bednar, D., 2020: FireProt^{DB}: Database of Manually Curated Protein Stability Data. *Nucleic Acids Research* 49: D319-D324
