# Supplementary material for: Biosensor and machine learning-aided engineering of an amaryllidaceae enzyme
Source: Nat Commun. 2024 Mar 7;15:2084. doi: 10.1038/s41467-024-46356-y (PMC10920890; doi:10.1038/s41467-024-46356-y)
Supplement: Supplementary file 3 — Reporting Summary [file 41467_2024_46356_MOESM3_ESM.pdf]

nature portfolio

Corresponding author(s): Simon d'Oelsnitz

Last updated by author(s): Feb 13, 2024

Reporting Summary

Nature Portfolio wishes to improve the reproducibility of the work that we publish. This form provides structure for consistency and transparency in reporting. For further information on Nature Portfolio policies, see our Editorial Policies and the Editorial Policy Checklist.

Statistics

For all statistical analyses, confirm that the following items are present in the figure legend, table legend, main text, or Methods section.

n/a	Confirmed	
☐	☒	The exact sample size (*n*) for each experimental group/condition, given as a discrete number and unit of measurement
☐	☒	A statement on whether measurements were taken from distinct samples or whether the same sample was measured repeatedly
☒	☐	The statistical test(s) used AND whether they are one- or two-sided<br>*Only common tests should be described solely by name; describe more complex techniques in the Methods section.*
☒	☐	A description of all covariates tested
☒	☐	A description of any assumptions or corrections, such as tests of normality and adjustment for multiple comparisons
☐	☒	A full description of the statistical parameters including central tendency (e.g. means) or other basic estimates (e.g. regression coefficient) AND variation (e.g. standard deviation) or associated estimates of uncertainty (e.g. confidence intervals)
☒	☐	For null hypothesis testing, the test statistic (e.g. *F*, *t*, *r*) with confidence intervals, effect sizes, degrees of freedom and *P* value noted<br>*Give P values as exact values whenever suitable.*
☒	☐	For Bayesian analysis, information on the choice of priors and Markov chain Monte Carlo settings
☒	☐	For hierarchical and complex designs, identification of the appropriate level for tests and full reporting of outcomes
☒	☐	Estimates of effect sizes (e.g. Cohen's *d*, Pearson's *r*), indicating how they were calculated

Our web collection on statistics for biologists contains articles on many of the points above.

Software and code

Policy information about availability of computer code

Data collection	Alphafold2 was used to create protein structure models. The Tecan Infinite M1000 plate reader was used to collect optical density and fluorescence measurements for all cell samples. The Thermo Vanquish HPLC system with chromeleon software (CHROMELEON7) was used to collect HPLC data. The Agilent 6530 Q-TOF system was used to collect LC/MS data. The Thermo Attune NxT device was used to collect flow cytometry data.
Data analysis	Python (version 3.8) was used for plotting data and estimating non-linear functions. Code used to generate bar plots and dose–response functions presented in this text is accessible at https://github.com/simonsnitz/plotting.<br>The FlowJo Software (version 10.8.1, FlowJo LLC) was used to calculate flow cytometry statistics.<br>GNINA1.0 was used for protein-ligand docking. The MutComputeX model as well as the input data of the norbelladine-4O-methyltransferase are available at https://github.com/danny305/MutComputeX. The KinTek Explorer software (version 11) was used for enzyme kinetics calculations.

For manuscripts utilizing custom algorithms or software that are central to the research but not yet described in published literature, software must be made available to editors and reviewers. We strongly encourage code deposition in a community repository (e.g. GitHub). See the Nature Portfolio guidelines for submitting code & software for further information.

Data

Policy information about availability of data

All manuscripts must include a data availability statement. This statement should provide the following information, where applicable:

- Accession codes, unique identifiers, or web links for publicly available datasets
- A description of any restrictions on data availability
- For clinical datasets or third party data, please ensure that the statement adheres to our policy

Protein sequence information was retrieved from the NCBI database: RamR, 3VVX_A; Nb4OMT, A0A077EWA5.1. Plasmid sequences relevant to this study can be found in Supplementary Table 7 and have been deposited in Addgene (216232, 216231). The Alfalfa caffeoyl coenzyme A 3'-O-methyltransferase (PDB: 1SUI) was used to assist with docking. Coordinates for the complex structure of Nb4OMTE36P/G40E/A53M with S-adenosyl-L-homocysteine (SAH) has been deposited in the Protein Data Bank (PDB) as 8UKE. Source data are provided with this manuscript.

Research involving human participants, their data, or biological material

Policy information about studies with human participants or human data. See also policy information about sex, gender (identity/presentation), and sexual orientation and race, ethnicity and racism.

Reporting on sex and gender	n/a
Reporting on race, ethnicity, or other socially relevant groupings	n/a
Population characteristics	n/a
Recruitment	n/a
Ethics oversight	n/a

Note that full information on the approval of the study protocol must also be provided in the manuscript.

Field-specific reporting

Please select the one below that is the best fit for your research. If you are not sure, read the appropriate sections before making your selection.

☒ Life sciences ☐ Behavioural & social sciences ☐ Ecological, evolutionary & environmental sciences

For a reference copy of the document with all sections, see nature.com/documents/nr-reporting-summary-flat.pdf

Life sciences study design

All studies must disclose on these points even when the disclosure is negative.

Sample size	Flow cytometry was used to collect data on at least 10,000 cells per sample. Each experimental condition was performed in biological triplicate, with the exception of LC/MS data. This sample size is in line the previous work in the field (see 10.1038/s42003-023-05363-3).
Data exclusions	Data were not excluded.
Replication	All experiments were performed with three biological replicates with the exception of the LC/MS experiments, which were performed in biological singlicate. These conditions are standard within the field. All attempts at replication were successful
Randomization	Samples were not randomized. Varying concentrations of inducer molecules and substrates were used, as described in the Methods section. The same experimental conditions were used for all samples that were compared to each other to minimize variation. This is standard in the field.
Blinding	Investigators were not blinded to experimental conditions, because similar strategies were required to be applied in all experiments.

Reporting for specific materials, systems and methods

We require information from authors about some types of materials, experimental systems and methods used in many studies. Here, indicate whether each material, system or method listed is relevant to your study. If you are not sure if a list item applies to your research, read the appropriate section before selecting a response.

Materials & experimental systems

n/a	Involved in the study
☒	☐ Antibodies
☒	☐ Eukaryotic cell lines
☒	☐ Palaeontology and archaeology
☒	☐ Animals and other organisms
☒	☐ Clinical data
☒	☐ Dual use research of concern
☒	☐ Plants

Methods

n/a	Involved in the study
☒	☐ ChIP-seq
☐	☒ Flow cytometry
☒	☐ MRI-based neuroimaging

Plants

Seed stocks	n/a
Novel plant genotypes	n/a
Authentication	n/a

Flow Cytometry

Plots

Confirm that:

☒ The axis labels state the marker and fluorochrome used (e.g. CD4-FITC).

☒ The axis scales are clearly visible. Include numbers along axes only for bottom left plot of group (a 'group' is an analysis of identical markers).

☒ All plots are contour plots with outliers or pseudocolor plots.

☒ A numerical value for number of cells or percentage (with statistics) is provided.

Methodology

Sample preparation	Bacterial samples were diluted 1:50 in 1x phosphate buffered saline solution. The Thermo Attune NxT instrument was used to measure the fluorescence of cell populations.
Instrument	Thermo Attune Nxt flow cytometer
Software	FlowJo Software (version 10.8.1, FlowJo LLC)
Cell population abundance	The cells were not sorted.
Gating strategy	n/a

☐ Tick this box to confirm that a figure exemplifying the gating strategy is provided in the Supplementary Information.
